# A remote monitoring system based on deep learning for real-time assessment of free flaps

**DOI:** 10.1371/journal.pone.0347343

**Published:** 2026-05-11

**Authors:** Xiaoyu Huang, Cong Cheng, Shiyou Ren, Ruifeng Fang, Fang Lu, Conger Wu, Zongyuan Jiang

**Affiliations:** 1 Department of Hand Surgery, People’s Hospital of Longhua, Shenzhen, China; 2 Department of Orthopedics, The Eighth Affiliated Hospital of Sun Yat-sen University, Shenzhen, China; 3 Department of Joint Surgery and Sports Medicine, Center for Orthopedic Surgery, Orthopedic Hospital of Guangdong Province, The Third Affiliated Hospital Southern Medical University, Guangzhou, China; Shuguang Hospital Affiliated to Shanghai University of Traditional Chinese Medicine, CHINA

## Abstract

**Purpose:**

Venous congestion is a major cause of postoperative free flap compromise, and early detection is crucial for improving flap salvage rates and patient outcomes. This study aimed to develop and validate a deep learning (DL)-integrated remote monitoring system with a smartphone application for real-time, quantitative assessment of free flaps, with a specific focus on the early detection of venous congestion.

**Methods:**

This diagnostic study was conducted at our institution. Patients aged 18–60 years who underwent free flap reconstruction between January 2019 and June 2025 were included. The study was divided into three phases: a 5-month model training phase for DL model development and internal validation, a 5-month external validation and clinical application phase, and a 4-month clinical comparison phase. The DL model was developed using TensorFlow Lite and a Flap Segmentation Network (FS-Net). Performance was evaluated through accuracy, sensitivity, specificity, area under the receiver operating characteristic curve (AUC), and clinical outcomes including time to detection and flap survival.

**Results:**

A total of 1649 photographs from 615 patients were analyzed in the development and validation of the DL classification model between January 2019 and February 2025. During model development, the model achieved an accuracy of 86.8%, sensitivity of 92.4%, specificity of 79.7%, and AUC of 0.88. Internal validation improved these metrics to 87.6%, 95.0%, 81.1%, and 0.92, respectively. External validation demonstrated the model’s generalizability, with an accuracy of 89.3%, sensitivity of 96.2%, specificity of 84.9%, and AUC of 0.93. The clinical application phase showed that the system had an overall accuracy of 92.16%, sensitivity of 95.18%, false-positive rate of 2.62%, and false-negative rate of 4.82%. A total of 113 patients were included in clinical comparison between March 2025 and June 2025. The remote monitoring group exhibited a trend towards a shorter mean time to congestion detection, higher flap survival rate, and shorter mean time to re-exploration, although these differences were not statistically significant.

**Conclusions:**

The DL-integrated remote monitoring system demonstrated high accuracy and reliability in detecting venous congestion. It provided an objective and real-time tool that may help reduce clinical burden and support timely intervention in free flap management. However, its impact on definitive clinical outcomes required further validation in larger studies.

## 1. Introduction

Free flap reconstruction is a cornerstone in reconstructive microsurgery. It enables the restoration of complex defects with high success rates ranging from 94% to 99% [1,2]. Despite significant improvements in surgical techniques, postoperative flap complications, such as vascular compromise, continue to occur in 3% to 10% of cases, potentially leading to partial or total flap failure if not detected and managed promptly [3,4]. Early identification of vascular compromise, particularly venous congestion, is critical, as timely intervention significantly improves salvage rates and clinical outcomes [5,6].

Conventional free flap monitoring relies heavily on frequent clinical assessments by trained personnel. These assessments evaluate parameters such as flap color, capillary refill, temperature, and turgor [7,8]. Although clinical assessments remain the criterion standard, they are inherently subjective and resource-intensive. This places a considerable burden on healthcare providers, especially during the critical early postoperative period when assessments may be needed hourly [9,10]. Moreover, inter-observer variability and the requirement for extensive clinical experience further limit the consistency and efficiency of this approach [11].

Recent advancements in artificial intelligence (AI), particularly in DL, have provided promising opportunities to enhance or potentially automate medical diagnostics [12,13]. In plastic and reconstructive surgery, AI has been applied to wound assessment, skin cancer detection, and complication prediction [14,15]. However, its integration into real-time, mobile-based flap monitoring systems remains limited. Previous studies have predominantly relied on non-portable devices or necessitated specialized hardware, thereby hindering its clinical application and translational potential [16,17].

The widespread availability of smartphones equipped with high-quality cameras and powerful processors presents a unique opportunity to develop accessible, cost-effective, and accurate monitoring tools [6]. Recent studies have demonstrated the feasibility of integrating DL models into mobile applications for flap monitoring, achieving high diagnostic accuracy and even outperforming human observers in some scenarios [12,13]. These systems can provide objective, quantitative assessments of flap perfusion status, reducing subjectivity and enabling continuous monitoring without proportional increases in clinical workload.

In this study, we aim to develop and validate a remote monitoring system that uses a DL-integrated smartphone application for the quantitative monitoring of free flaps, with a focus on the early detection of venous congestion. By leveraging a large dataset of clinical images and state-of-the-art DL techniques, we attempt to create a reliable, non-invasive tool that can assist clinicians in early detection of flap compromise, ultimately improving patient safety and clinical outcomes.

## 2. Materials and methods

### 2.1. Study population

This was a diagnostic study that had been approved by the Institutional Committee on Ethics in our institution (Approval No.2018−123). The study was conducted and reported in strict accordance with the Standards for the Reporting of Diagnostic Accuracy Studies (STARD statement). Its aim was to develop and validate a remote monitoring system integrated with a DL model for quantifying the monitoring of venous congestion in free flaps and achieving clinical translation.

The study population included patients who underwent free flap reconstruction aging from 18 to 60 years old between January 2019 and June 2025. Exclusion criteria included those who received local flaps, pedicled flaps, skin grafts, intraoral flaps, buried flaps, and de-epithelialized flaps. The primary endpoint was flap survival at a standardized postoperative follow-up of 90 days. Flap survival was confirmed during scheduled outpatient clinic visits at 1 month and 3 months postoperatively, where flaps were assessed clinically and photographically by the surgical team. For patients unable to return, flap status was confirmed by structured telephone interview and review of photographs sent by the patient.

### 2.2. Data collection

Retrospective photographs were taken using a standardized camera-based monitor (Logitech Brio 4K Pro Webcam). The monitor was set to a resolution of 3840 x 2160 pixels (4K UHD) and featured a 1/2.8-inch CMOS sensor with a fixed focal length of 2.8 mm (f/2.0 aperture). To ensure consistent and reproducible lighting critical for color assessment, a dimmable LED flat-panel light (Phive P100 Medical Grade LED Light) was used. Lighting parameters were standardized with color temperature at 5500K (daylight-equivalent) to provide neutral white light, and brightness calibrated to 5500 ± 500 lux at the flap surface (measured with a digital lux meter, Extech SDL400). The light source was positioned vertically above the surgical site at a fixed distance to minimize shadows. Photographs were acquired from five consistent orientations (frontal, superior, inferior, left lateral, and right lateral) at a consistent distance to ensure accurate identification of flap margins and surrounding areas. Photographs were taken every 1 hour during the patients’ stays.

All microsurgical reconstructive procedures were performed by 3 board-certified plastic surgeons specializing in microsurgery, each completing over 60 reconstructive microsurgeries annually as attending surgeons with more than 5 years of experience. Consistent with existing study [1,4], clinical observation was regarded as the gold standard for assessment, including skin color, temperature of the flap’s skin island (compared with the surrounding recipient site), capillary refill time, and skin turgor. Assessments were conducted hourly by trained nurses and every 2 hours by resident physicians. If congestion was suspected, a pinprick test was performed. If two consecutive pinprick tests (conducted 1 hour apart) were positive results, or if a positive result occurred following anticoagulant administration, surgical exploration was arranged.

The study period was divided into three distinct phases with a strict patient-level cohort separation to ensure rigorous evaluation of model generalizability and address the statistical dependency of serial images from the same patient. The first phase (from January 2019 to May 2022) was the retrospective classification model training phase, during which the acquired photographs were utilized for the development and internal validation of the DL model. For this phase, a patient-level 10-fold cross-validation was employed. All photographs belonging to a single patient were kept within the same fold (either training or internal validation for that iteration), thus the model was never validated on images from a patient it was trained on in the same run.

The subsequent phase (from June 2022 to February 2025) formed the prospective external validation and clinical application phase, and the collected photographs were employed to validate the effectiveness of the developed model. The external validation set was constructed from patients temporally separate from the development cohort and not involved in model training. To mitigate over-representation of individual patients and obtain a performance estimate robust to within-patient variation, we standardized image selection by randomly choosing two photographs per patient to prioritize different postoperative time points and frontal/lateral angles. This balance provided stability by multiple data points per patient while avoiding statistical inflation from including all serial images. During the two phases, patients were categorized into two groups. The normal group included patients who underwent free flap reconstruction without any clinical suspicion of flap venous congestion. Patients with suspected venous congestion confirmed by venous thrombosis during re-exploration were included in congestion group. Within this group, patients with flap failure or severe necrosis requiring secondary reconstruction were classified as “flap failure.” Cases with suspected congestion but no re-exploration or negative venous occlusion results were excluded.

The final phase (from March 2025 to June 2025) was designated as a non-randomized observational study, which objective was to compare the outcomes of two monitoring techniques: conventional methods (verbal reports and visual assessment) and the remote monitoring system. The group allocation was based on practical clinical workflow arrangements, such as ward equipment configuration and clinicians’ decision-making preferences. This prospective cohort constituted a wholly independent test at the patient level that simulated a real-world deployment scenario. The outcome measures included flap survival rate, time to detection of threatened flaps, and time to re-exploration (the time interval between the first notification of vascular compromise and the initiation of re-exploration).

### 2.3. Data processing

**Fig 1:**
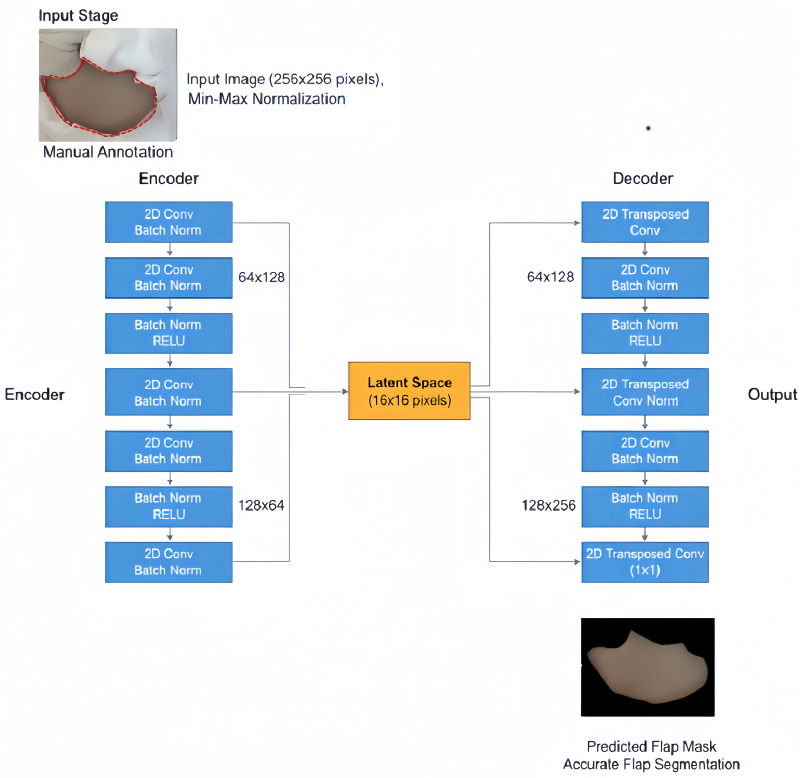
Flap Segmentation Network (FS-Net) architecture.

The custom CNN architecture, Flap Segmentation Network (FS-Net) was chosen to accurately and automatically identify the flap region in captured photographs [12]. Manual annotation along the flap margins was performed on recruited photographs depicting flap appearance. Photographs with well-defined flap edges were selected as suitable for model construction and training. The encoder stage employed successive 2-dimensional (2D) convolutional layers with batch normalization and ReLU (Rectified Linear Unit) activation functions to compress input images (256x256 pixels) into a compact 16x16-pixel representation. Mirroring this structure, the decoder utilized 2D transposed convolutional layers to perform upsampling and restore the original spatial resolution. All input images were uniformly resized to 256x256 pixels, with pixel values scaled using min-max normalization (Fig 1).

The collected photographs were labeled into two categories: normal and congestion. Labeling was performed based on clinical evaluation by two independent and experienced microsurgeons. Although inter-rater disagreements were infrequent, they were resolved through discussion to achieve consensus in all cases. To ensure label quality and minimize ambiguity in the training data, we established pre-defined exclusion criteria during the labeling process. Photographs were excluded from model training if they met any of the following objective criteria: (1) the presence of obstructive elements (e.g., surgical dressing, significant shadow, or blood clot) covering more than 20% of the flap area; (2) severe image artifacts (e.g., motion blur, lens flare) that substantially degraded visual clarity; (3) the presence of confounding pathologies (e.g., large hematoma, active bleeding, or wound exudate) that, in the unanimous judgment of the two microsurgeons, made the reliable distinction between normal, congested, or ischemic tissue impossible. Cases where the two surgeons disagreed on the classification label but agreed that the image was interpretable were resolved through consensus discussion and retained in the dataset.Photographs of normal and congested flaps were collected during the model development, and used as training data for TensorFlow Lite, which was a lightweight, open-source DL framework designed for mobile and embedded devices. It was capable of identifying designated objects within images, classifying them, and calculating the probability that the recognized object belongs to each specified category. TensorFlow Lite, built upon TensorFlow, supported model quantization and optimization, enabling efficient inference on resource-constrained devices. We utilized TensorFlow Lite Model Maker (version 2.13.0, Google LLC) to perform semi-supervised learning from the training data. Upon completing the image analysis, the tool generated the corresponding DL model code. The output data was consist of prediction labels and their associated probability distributions. The training was conducted on a workstation (Dell Inc.) equipped with an Intel Core i7-11700K processor, 32 GB memory (3200 MHz DDR4), and an NVIDIA RTX 3080 GPU with 10 GB VRAM. The maximum number of training epochs was set to 30.

Key hyperparameters for model training were as follows: The model was optimized using the Adam optimizer with an initial learning rate of 0.001, beta1 = 0.9, and beta2 = 0.999. Binary cross-entropy was employed as the loss function. Training was performed with a batch size of 32. To mitigate overfitting, L2 weight regularization (λ = 0.0001) was applied to the convolutional layers, and a dropout rate of 0.5 was used in the final fully connected layer. Although the maximum epoch was 30, an early stopping callback was implemented to halt training if the validation loss did not improve for 5 consecutive epochs, with model weights restored to the point of best validation performance.

### 2.4. Development of remote monitoring system and assessment of clinical outcomes

**Fig 2:**
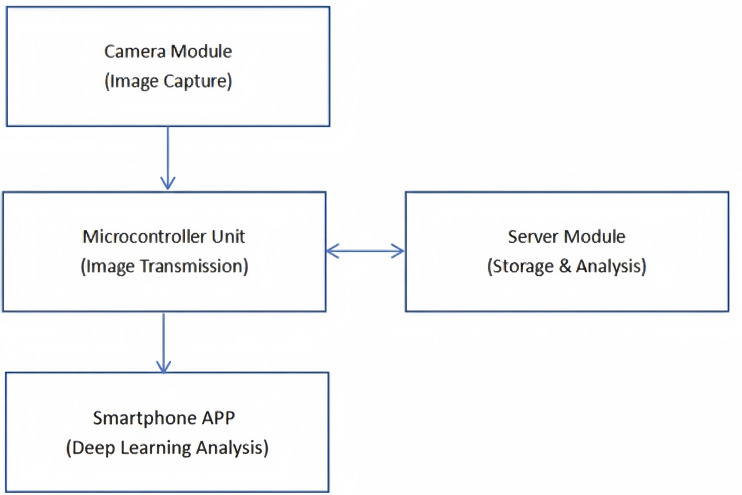
The remote monitoring system architecture.

The remote monitoring system has several key components that work collaboratively to provide real-time observation of the free flap’s condition and offer accurate diagnostic support. These components were consist of a camera-based monitor, a data transmission module, a server system, and a smartphone application (“DLscope” App)(Fig 2).

The camera-based monitor is designed to capture high-resolution images of the surgical site at regular intervals to track the flap’s condition. This monitor allows to detect early venous congestion. It has high-resolution imaging to detect subtle changes, and low-light capability with LED for clear images in dark environment to ensure comprehensive monitoring. The camera captures images every 2 seconds, enabling continuous observation of the surgical site, thus ensuring that critical changes are not missed.

The Microcontroller Unit (MCU) is responsible for coordinating image capture at specified intervals, and performing basic image processing tasks such as compression to reduce file size. This helps improve transmission efficiency and reduce data transfer time. It also communicates with the camera and the server via a Wi-Fi module, which transmits the data wirelessly.

**Fig 3:**
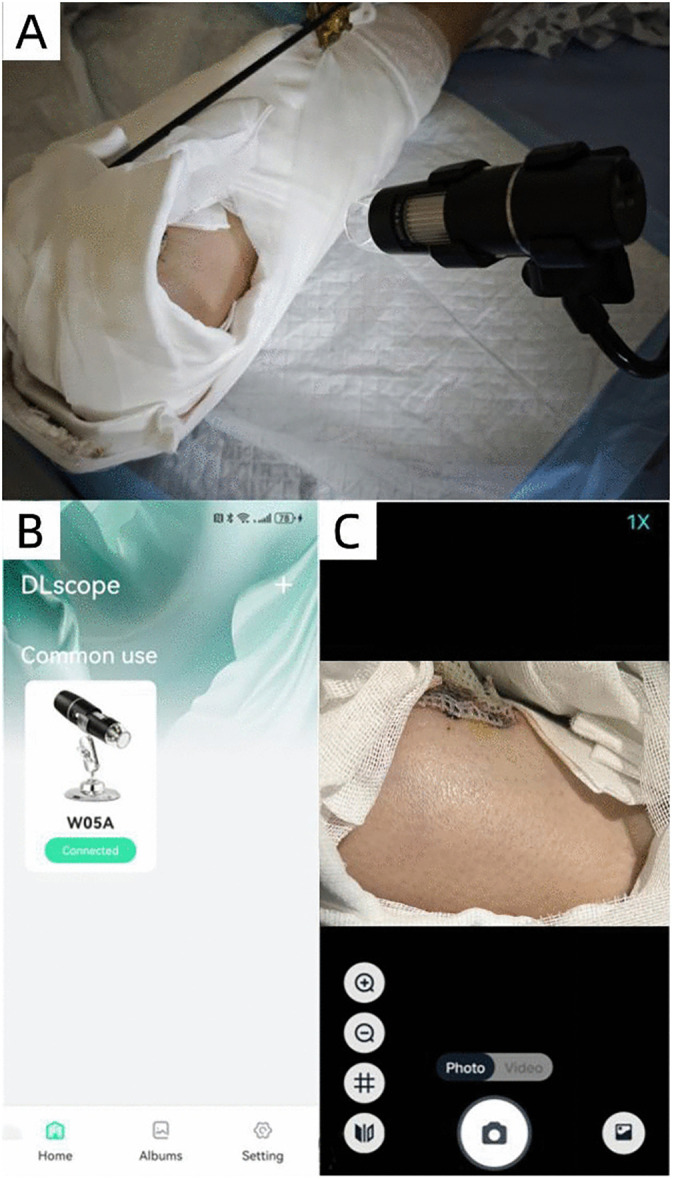
Real-time assessment of free flap by remote monitoring system. (A) A camera-based monitor was fixed at the bedside. (B,C) The interface of the‘’DLscope” application in the smartphone.

**Fig 4:**
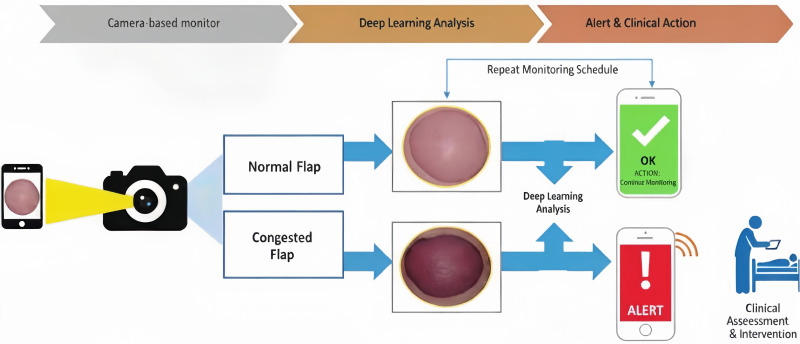
Schematic workflow of the free flap remote monitoring. A camera-based monitor was fixed at the bedside where the flap could be clearly observed. The monitor could take pictures and sent them to the‘’DLscope” application in the smartphone automatically at regular intervals. The application would automatically identify the flap area from the images and assess its perfusion status. If there were no problems, the process would be repeated. If venous congestion was detected, the pre-arranged medical staff would be called to directly assess the status of the flap.

**Fig 5:**
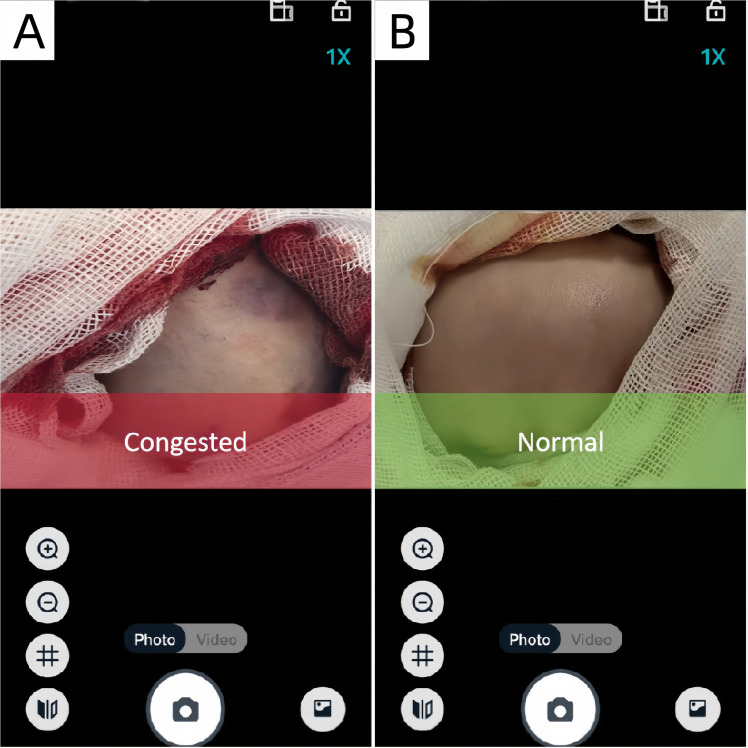
Identification of venous congestion by remote monitoring system. (A) The message stating “Congested” in the screen is displayed. (B) The message stating “Normal” in the screen is displayed.

The server system plays a crucial role in storing, managing, and processing the images and data sent from the camera and MCU. It serves as the central hub, organizing the images in a time-series database for easy retrieval and analysis. The smartphone application could provide a user interface to display real-time images and data from the monitoring system (Fig 3). It is designed with a workflow to automatically capture flap photographs, perform segmentation on the images, assess congestion status, and repeat the process if results are normal (Fig 4). The prediction result is displayed on-screen. To enhance clinical utility and ensure prompt notification, a multi-modal alert system was implemented. If the computed probability of venous congestion exceeds 50%, the prediction label turns red, and an message stating “Congested” in the screen is displayed. If the computed probability of venous congestion is less than 50%, the message stating “Normal” is displayed (Fig 5).

### 2.5. Statistical analysis

The photographs of free flaps were imported into the “DLscope” app by a researcher who were unaware of the patients’ clinical grouping, flap conditions, and prognosis, and recorded the classification labels and probability distributions. TensorFlow Lite Model Maker (version 2.13.0, Google LLC) was used to evaluate the training and validation accuracy of the DL model. Sensitivity, specificity, and the area under the receiver operating characteristic curve (AUC) were calculated with SPSS 29.0.2.0 (IBM Corp.).

For patient demographics, categorical variables were analyzed via X^2^ or Fisher’s exact tests. Continuous variables were first tested for normality using the Shapiro-Wilk test. Variables with a Shapiro-Wilk test P-value >0.05 were considered to follow a normal distribution and analyzed using independent samples t-tests, while those with P ≤ 0.05 (non-normal distribution) were analyzed using Mann-Whitney U tests. Independent samples t-tests compared congestion probabilities between normal and congested groups. The paired X^2^ test was adopted to compare the consistency between the classification results of the model and clinical observers. In the clinical comparison phase, pre-specified stratified subgroup analyses were performed by flap type to address the limited statistical power. A P-value < 0.05 was considered statistically significant.

## 3. Results

### 3.1. DL model performance

The DL model was systematically trained and validated using standardized flap photographs, and its performance metrics were rigorously assessed across the model development, internal validation, and external validation stages (Table 1).

**Table 1:** Data characteristics of the development and validation of the DL model.

	No. Of Patients	No. Of Photographs		Accuracy(%)	Sensitivity(%)	Specificity(%)	FNR(%)	FPR(%)	AUC
		Normal	Congested						
Development	280	256	86	86.8	92.4	79.7	7.22	6.32	0.88
Internal Validation				87.6	95.0	81.1	6.50	6.03	0.92
External Validation	231	434	28	89.3	96.2	84.9	5.32	3.17	0.93

FNR = false-negative rate, FPR = false-positive rate, AUC = area under the receiver operating characteristic curve.

**Fig 6:**
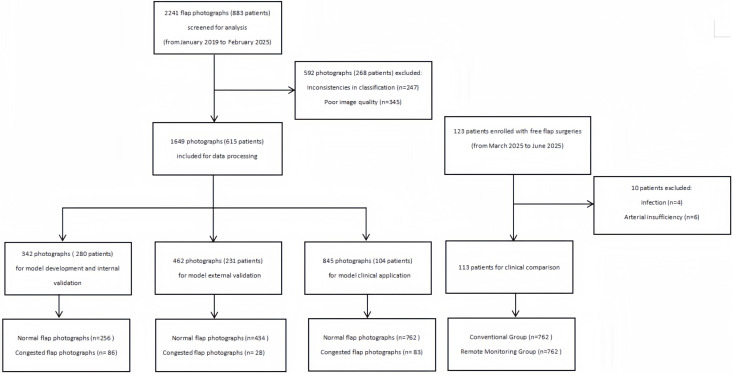
Participant Recruitment Flowchart.

**Fig 7:**
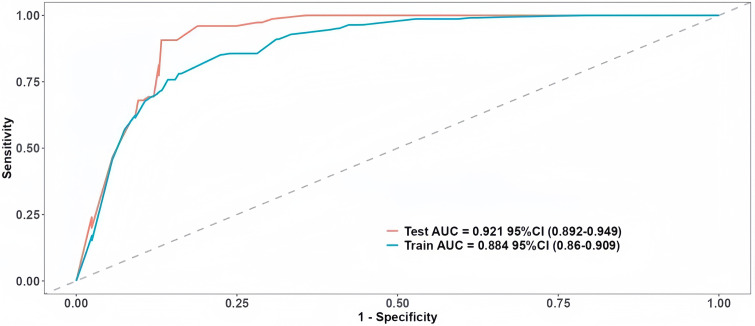
ROC for Development and Internal Validation of the DL Model.

**Fig 8:**
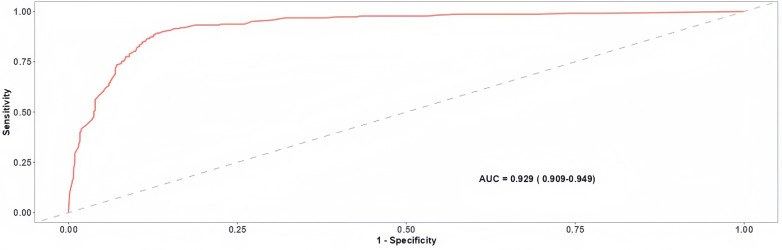
ROC for External Validation of the DL Model.

During the model development phase, a total of 342 photographs were used, including 256 normal and 86 congested flap photographs from 280 patients (Fig 6). The model achieved a development accuracy of 86.8% with a sensitivity of 92.4% and specificity of 79.7%. The AUC was 0.88. Internal validation was performed on the same dataset via 10-fold cross-validation, which yielded improved performance, with an accuracy of 87.6%, sensitivity of 95.0%, specificity of 81.1%, and an AUC of 0.92, indicating robust learning and minimal overfitting (Fig 7). For external validation, we tested the model on a completely independent patient cohort. A total of 462 photographs including 434 normal and 28 congested flap photographs from 231 patients were used for external validation, with two images randomly selected per patient to ensure representativeness. The model demonstrated strong generalizability, achieving an accuracy of 89.3%, sensitivity of 96.2%, specificity of 84.9%, and an AUC of 0.93, confirming the model’s high diagnostic performance and reliability across independent cohorts (Fig 8).

### 3.2. Clinical application

During the clinical application, a final cohort of 104 patients with 845 photographs was analyzed using the “DLscope” application, including 92 patients with normal flaps and 12 with venous congested flaps. The model correctly identified 725/762 (95.1%) normal flap photographs and 79/83 (94.0%) congested flap photographs. This corresponded to an overall accuracy of 92.16%, with a sensitivity of 95.18%. False-positive and false-negative rates were 2.62% and 4.82%, respectively (Table 2).

**Table 2:** Clinical application of the DL model.

Variable	Normal Group	Congested Group	P value
No. of patients, n	92	12	
No. of photographs, n	762	83	
Normal flap	742	4	<0.001
Congested flap	20	79	

### 3.3. Clinical comparison of conventional and remote monitoring groups

A total of 123 patients were initially enrolled, with 10 excluded due to venous congestion caused by non-venous factors (4 patients with infection, and 6 patients with arterial insufficiency). 113 free flap patients were included with 56 free flaps in the conventional monitoring group and 57 free flaps in the remote monitoring group between March 2025 and June 2025. Detailed baseline characteristics of the two groups were summarized in Table 3. There were no statistically significant differences in demographic and clinical variables between the two groups (all P > 0.05), indicating that the baseline conditions were well-balanced and comparable.. The flap types, including deep inferior epigastric perforator (DIEP), anterolateral thigh (ALT) flaps, radial forearm (RF) flap, latissimus dorsi (LD) flap, radial artery superficial palmar branch (RASPB) flap, and toe pulp (TP) flap were distributed similarly between the two groups.

**Table 3:** Baseline demographic and clinical characteristics of conventional and remote monitoring groups.

Variable	Conventional Group	Remote Monitoring Group	P value
No. of patients, n	56	57	
Age, mean (SD)	35.66 ± 10.34	39.37 ± 12.68	0.09
Gender			
Male, n(%)	41 (73.2%)	36 (63.2%)	0.25
Female, n(%)	15 (26.8%)	21 (36.8%)	
BMI, mean (SD)	24.3 ± 3.1	23.8 ± 2.9	0.42
Preoperative hemoglobin (g/L), mean (SD)	135.2 ± 12.8	132.6 ± 13.5	0.31
Preoperative Serum Albumin (g/L), mean (SD)	38.5 ± 4.2	37.9 ± 4.5	0.50
Preoperative ASA Classification (n, %)			0.81
Grade I-II	48 (85.7)	49 (86.0)	
Grade III-IV	8 (14.3)	8 (14.0)	
Surgery duration (min), mean (SD)	215.4 ± 42.6	223.1 ± 38.9	
No. of flaps, n	56	57	
Flap type			
DIEP, n(%)	15 (26.8%)	14 (24.6%)	0.83
ALT, n(%)	21 (37.5%)	23 (40.4%)	0.77
RF, n(%)	5 (8.9%)	8 (14.0%)	0.41
LD, n(%)	6 (10.7%)	3 (5.3%)	0.22
RASPB, n(%)	6 (10.7%)	5 (8.8%)	0.75
TP, n(%)	3 (5.4%)	4 (7.0%)	0.10
Comorbidities, n	11	9	
smoking, n(%)	8 (14.3%)	6 (10.5%)	0.41
diabetes, n(%)	2 (3.6%)	2 (3.5%)	0.96
POVD, n(%)	1 (1.8%)	0 (0%)	0.22
CHD, n(%)	1(1.8%)	1 (1.7%)	0.99

BMI = Body Mass Index, SD = Standard Deviation, ASA = American Society of Anesthesiologists, DIEP = deep inferior epigastric perforator, ALT = anterolateral thigh, RF = radial forearm, LD = latissimus dorsi, RASPB = radial artery superficial palmar branch, TP = toe pulp, POVD= peripheral occlusive vessel disease, CHD= chronic heart disease.

Baseline continuous variables were assessed for normality with results summarized in S1 Table. Age, body mass index (BMI), preoperative hemoglobin, and operation duration were normally distributed, while time to detection of congested flaps and time to re-exploration exhibited a non-normal distribution. Corresponding parametric (independent samples t-test) and non-parametric (Mann-Whitney U test) tests were subsequently applied for intergroup comparisons of these continuous variables.

In both the conventional and remote monitoring groups, 9 congested flaps were identified, respectively. The time to detection of abnormal signs indicating venous congestion was analyzed. On average, the onset of abnormal signs occurred at 19.4 hours (range: 2.0 to 36.5 hours) after surgery in the conventional group and at 14.6 hours (range: 1.8 to 26.7 hours) in the remote monitoring group. The remote monitoring group exhibited a trend towards earlier detection of congested flaps, however the difference was ont statistically significant (p = 0.14).

One of the most critical findings in this study was the time interval between the first recognition of venous congestion and the initiation of re-exploration. In the conventional group, the average time interval was 3.5 hours (range: 1.0 to 7.7 hours). while in the remote monitoring group, the average time interval was 1.6 hours (range: 1.0 to 4.2 hours), indicating no significant difference (p = 0.08).

In the conventional group, 5 out of the 9 congested flaps were successfully salvaged after re-exploration. The remaining 4 flaps were not salvageable, experiencing complete necrosis and requiring flap abandonment. In contrast, in the remote monitoring group, 7 out of the 9congested flaps were successfully salvaged. The remaining 1 flap experienced complete necrosis. Overall, 52 out of 56 flaps (91.1%) survivedin the conventional monitoring group, while 55 out of 57 flaps (98.2%) survived in the remote monitoring group. The difference was not statistically significant (p = 0.11), although a trend towards higer flap survival rate was detected in the remote monitoring group (Table 4).

**Table 4:** Free flap outcomes of conventional and remote monitoring groups.

Variable	Conventional Group	Remote Monitoring Group	P value
No. of Patients, n	56	57	
No. of Congested Flaps, n(%)	9(16.1)	9(16.1)	
Time to Detection of Congested Flaps (hr)	19.4 (2.0-36.5)	14.6 (1.8-26.7)	0.14
Time to Re-exploration (hr)	3.5 (1.0-7.7)	1.6 (1.0-4.2)	0.08
Flap Survival, n(%)	52 (92.8%)	55 (96.5%)	0.11

The subgroup analysis by flap type revealed heterogeneous trends favoring the remote monitoring system (Table 5). Notably, in ALT flaps, the remote monitoring group showed a trend toward higher flap survival (95.7% vs. 85.7%, p = 0.06) and a substantially higher successful salvage rate (80.0% vs. 50.0%) compared to the conventional group. In DIEP flaps, the remote monitoring group achieved 100% survival (vs. 86.7% in the conventional group, p = 0.12) and 100% salvage rate (vs. 50.0%). No meaningful differences emerged in other less common flap subtypes, likely due to small subgroup sample sizes.

**Table 5:** Stratified Subgroup Analysis of Clinical Outcomes by Flap Type.

Flap Type	Group	No. of Patients	No. of Congested Flaps	Time to Detection (hr)	Time to Re-exploration (hr)	Flap Survival, n(%)	Successful Salvage Rate (%)	P Value (Survival)
DIEP	Conventional	15	2	21.3 (4.2-35.8)	3.8 (1.2-7.5)	13 (86.7)	50.0 (1/2)	0.12
	Remote	14	2	15.7 (2.1-25.3)	1.7 (1.0-3.9)	14 (100.0)	100.0 (2/2)	
ALT	Conventional	21	4	18.9 (2.0-36.5)	3.6 (1.0-7.7)	18 (85.7)	50.0 (2/4)	0.06
	Remote	23	5	13.8 (1.8-24.6)	1.5 (1.0-4.2)	22 (95.7)	80.0 (4/5)	
RF	Conventional	5	1	16.5 (3.1-28.7)	2.9 (1.1-5.3)	5 (100.0)	100.0 (1/1)	>0.99
	Remote	8	1	12.3 (2.5-20.1)	1.4 (1.0-2.8)	8 (100.0)	100.0 (1/1)	
LD	Conventional	6	1	23.1 (5.4-32.2)	4.1 (1.5-6.8)	5 (83.3)	0.0 (0/1)	0.48
	Remote	3	0	17.2 (3.8-22.5)	–	3 (100.0)	–	
RASPB	Conventional	6	1	17.8 (2.8-30.1)	3.2 (1.0-6.1)	6 (100.0)	100.0 (1/1)	>0.99
	Remote	5	1	13.1 (2.0-21.4)	1.3 (1.0-2.5)	5 (100.0)	100.0 (1/1)	
TP	Conventional	3	0	19.2 (4.5-27.3)	–	3 (100.0)	–	>0.99
	Remote	4	0	14.5 (2.2-23.8)	–	4 (100.0)	–	
Total	Conventional	56	9	19.4 (2.0-36.5)	3.5 (1.0-7.7)	52 (92.8)	55.6 (5/9)	0.11
	Remote	57	9	14.6 (1.8-26.7)	1.6 (1.0-4.2)	55 (96.5)	77.8 (7/9)	

DIEP = deep inferior epigastric perforator, ALT = anterolateral thigh, RF = radial forearm, LD = latissimus dorsi, RASPB = radial artery superficial palmar branch, TP = toe pulp.

## 4. Discussion

Venous congestion, one of the most common causes of postoperative flap compromise, remains a critical threat to clinical outcomes, as delayed detection can lead to flap necrosis, fistula formation, or even complete failure [5,9]. Traditional free flap monitoring relies on hourly clinical observations of flap color, turgor, and capillary refilling by nurses and resident surgeons. This process is inherently subjective, labor-intensive, and prone to variability due to observer experience, fatigue, or emotional state [1,10]. The requirement for frequent in-person assessments also placed a significant burden on healthcare staffing [7]. The development and validation of our DL-integrated remote monitoring system for quantitative monitoring of free flap venous congestion represented a significant step toward intelligent and automated postoperative care in reconstructive microsurgery. This system had high performance metrics of an AUC of 0.92 in internal validation and 0.93 in external validation. The results affirmed its technical viability and diagnostic reliability. It also demonstrated earlier detection of venous congestion, shorter time to re-exploration, and higher flap survival rate in the remote monitoring group when compared with the conventional group, however there were no significant differences. This may be attributed to the relatively small sample size, with only 57 patients in the remote monitoring group and 56 in the conventional group.

These results of our study were highly consistent with recent advancements in AI-assisted flap monitoring. Hsu et al. [13] reported similar outcomes with an iOS-based application that achieved an AUC of 0.99 and enabled probabilistic assessment of venous congestion. Likewise, Kim et al. [12] developed a fully automated monitoring system, FLAPMATE, which used DenseNet121 for anomaly detection and attained an AUC of 0.96. Our model built upon these foundations. It incorporated real-time predictive capabilities and offering a user-friendly interface that requires no specialized hardware. These attributes were essential for facilitating clinical adoption, particularly in resource-limited settings.

A key strength of our DL-integrated system is its capacity to convert subjective visual cues into continuous, numerical values. Traditional monitoring, though considered the criterion standard, is inherently qualitative and vulnerable to inter-observer variability [9]. Factors such as clinician experience, ambient lighting, and even fatigue can influence perceptual judgments [10]. By contrast, AI models can analyze pixel-level patterns and texture features that are imperceptible to the human eye, enabling earlier and more consistent detection of venous congestion. In our study, the system flagged congestion with high probability before clinical confirmation in several cases. This lead time is clinically meaningful, as the success of flap salvage declines rapidly with delayed detection [1].

Clinical data underscores the pivotal role of timely detection in flap salvage. Chen et al. [18] analyzed 1,142 free flap procedures and found that flaps that were re-explored within 6 hours of vascular compromise had a salvage rate of 83%, compared to only 33% when intervention was delayed beyond 12 hours. This temporal relationship was particularly pronounced in hand and foot reconstructions, where smaller vessel diameters and higher mechanical stress increase susceptibility to thrombosis. For instance, in a series of lower extremity reconstructions, Bui et al. [19] reported that free flaps identified as threatened within 24 hours of surgery had a 75% salvage rate, whereas delays beyond 48 hours reduced this to 20%, suggesting that the window for successful re-intervention narrowed rapidly, with each hour of delay incrementally reducing the likelihood of restoring perfusion and preventing flap necrosis. We observed that flap venous congestion probability peaked immediately postoperatively and gradually declined, stabilizing after 72 hours. This capacity for longitudinal tracking allowed clinicians to distinguish normal postoperative evolution from pathological changes. Thus it helped reduce false alarms and reinforce decision-making.

Another promising feature of our system is its ability to capture physiological trends over time. Free flaps rely on immediate revascularization to sustain cellular metabolism [20]. Kerrigan et al. [21] demonstrated that skeletal muscle, a common component of flaps used in hand and foot reconstruction, could tolerate ischemia for approximately 4–6 hours before irreversible cellular damage occurs. Skin and subcutaneous tissues exhibit greater resilience, with a critical ischemia time extending to 6–8 hours, though this window narrows significantly in the presence of venous occlusion due to edema and microvascular collapse. Thus, the time to detection of a congested flap directly influences whether congestive insult remains reversible, making early identification paramount.

Even after timely detection, delay in re-exploration results in ischemic damage. Optimizing flap survival requires minimizing the time to exploration through robust monitoring and rapid response systems. Shen et al. [1] revealed that of the 4,403 free flaps included in the analysis, 35.9% experienced total failure, while 64.6% were successfully salvaged. The average time for successful salvage was 30.8 hours after vascular compromise detection, compared to 51.5 hours for flaps that failed despite intervention. In addition, this study found that venous and arterial issues were the most common causes of failure, accounting for 59.5% and 27.9% of the cases, respectively. The majority of vascular compromise occurred even earlier than 72 hours, and intensive monitoring was most valuable during the first 48 hours. After this window, both the risk of compromise and the chance of successful salvage significantly decreased sharply.

Moreover, the integration of DL and mobile technology significantly enhances practicality and scalability. Earlier systems frequently required fixed imaging conditions or dedicated devices [7]. In contrast, our application operated on standard smartphones and accommodated variations in background, illumination, and camera type. This flexibility was a substantial advantage over previous attempts. For example, the system by Kiranantawat et al. [6] relied on rigid algorithms for pixel-intensity comparisons and performed poorly under suboptimal conditions. The use of contemporary DL frameworks, specifically optimized for mobile deployment, ensures that inferences are both rapid and computationally efficient.

Several directions should guide future research. First, the model was currently restricted to detecting venous congestion and could not identify arterial insufficiency, which was a gap shared by many existing AI tools [13]. Arterial compromise, though less common than venous congestion, requires equally urgent intervention. The visual signs of arterial ischemia, such as pallo, loss of turgor and delayed capillary refill, are different fundamentally from those of venous congestion. Therefore, the model in its present form could not provide a complete assessment of vascular compromise. Future iterations should incorporate training data from flaps with arterial insufficiency to expand its diagnostic capabilities. Second, multi-center validation is essential to establish the model’s generalizability. While its performance has been demonstrated within our institution’s standardized context, the robustness across diverse hospital settings, varied imaging equipment, and heterogeneous clinical workflows remains unproven. Variability in these factors could directly impact image characteristics and thus model performance. Therefore, prospective validation across a range of clinical environments is the critical next step to confirm its practical utility and translational potential [22]. Third, explainable AI techniques, such as Grad-CAM (Gradient-weighted Class Activation Mapping), will be crucial for enhancing clinical trust and adoption. These techniques can generate visual heatmaps that highlight the image regions most influential to the model’s prediction of congestion. Such visual explanations would greatly enhance clinician understanding, trust and acceptance, facilitating a collaborative human-AI decision-making process [23]. Furthermore, combining image-based analysis with multimodal data, such as temperature, capillary refill time, or even near-infrared spectroscopy, could create a more comprehensive monitoring system [24,25]. Finally, cost-effectiveness analyses should be conducted to evaluate whether AI-assisted monitoring reduces reoperation rates, or lowers overall healthcare costs [26].

Although our DL-integrated system demonstrated high diagnostic accuracy, the clinical comparison phase revealed only trend-level improvements in time to detection, time to re-exploration, and flap survival rates without statistical significance. It is crucial to avoid overstating these findings as evidence of clinical superiority. The lack of statistically significant differences may be attributed to the inherent limitations of this preliminary comparative study, which was designed primarily to evaluate feasibility. Given the high baseline flap survival rate (>90%) and the relatively small sample size, the study had limited statistical power to detect a clinically meaningful improvement. Future studies with larger, prospectively enrolled populations are therefore necessary to adequately determine whether these observed trends translate into statistically and clinically significant benefits. The primary value of this phase lied in demonstrating the feasibility and safety of integrating the remote monitoring system into the clinical workflow, and in generating promising preliminary data to inform the design of more definitive, large-scale trials.

Several limitations must be acknowledged. First, while we included variable imaging conditions, the training cohort was ethnically homogeneous, and the model’s performance in patients with darker skin tones remained untested. This was a critical limitation, as skin color variation could affect the model’s ability to detect subtle discoloration. Our model was developed primarily on a population from a single geographic region, which limited the diversity of skin tones in the dataset. Since the visual detection of venous congestion relies on assessing color changes, these signs may present with different contrast or subtleness across the spectrum of skin pigmentation. Consequently, a model trained predominantly on lighter skin tones may not generalize optimally to patients with darker skin, potentially introducing diagnostic bias and compromising equity in care. Second, there was an image-level dependency inherent in using multiple photographs from a small number of patients for model training and validation. Although we used patient-level splits and selected two representative images per patient for external validation to reduce correlation, the model’s performance may still be overestimated due to intra-patient image similarity, which could affect its generalizability to new patients with distinct clinical characteristics. Third, it was a non-randomized observational study with a limited sample size. The non-random allocation based on clinical workflow introduced the potential for selection bias and unmeasured confounding. The sample size provided insufficient statistical power to detect statistically significant differences in clinical outcomes, despite observed favorable trends. Therefore, these clinical findings should be viewed as preliminary and hypothesis-generating. Another challenge was image quality control. Extreme motion artifacts, blur, or shadowing could compromise prediction accuracy. Integrating automated quality checks, which prompted users to retake unclear images, could mitigate this issue. Additionally, although the system demonstrated high specificity, false positives still occurred in cases with bruising or hematoma, which could mimic congestion [3]. Such scenarios underscore the importance of maintaining human oversight and contextual interpretation. Moreover, ethical and practical considerations also warrant discussion. AI tools should augment, not replace, clinical judgment [14]. Our system was designed as a sentinel to detect potential issues while preserving the clinician’s role in final decision-making. This human-AI collaboration was essential both for patient safety and professional accountability [27]. Moreover, successful implementation should necessitate training for nursing and surgical teams to foster trust and ensure appropriate use.

## 5. Conclusions

Our DL-integrated system representsed a reliable and scalable tool for the objective, real-time assessment of free flap venous congestion. While encouraging trends towards earlier detection and higher survival were observed, these clinical benefits required confirmation in larger, adequately powered studies. The system has the potential to standardize postoperative monitoring and support earlier intervention. As AI continues to evolve, its thoughtful integration into clinical practice will play an increasingly vital role in advancing the safety and efficacy of reconstructive microsurgery.

## Supporting information

S1 Table Normality Test Results for Continuous Variables (Shapiro-Wilk Test). (DOCX)

S1 File Trial Study Protocol. (DOCX)

S2 File TREND Statement Checklist. (DOCX)
