# Supplementary material for: A remote monitoring system based on deep learning for real-time assessment of free flaps
Source: PLoS One. 2026 May 11;21(5):e0347343. doi: 10.1371/journal.pone.0347343 (PMC13160306; doi:10.1371/journal.pone.0347343)
Supplement: S1 Table — (DOCX) [file pone.0347343.s001.docx]

**Table S1. Normality Test Results for Continuous Variables (Shapiro-Wilk Test)**

| Continuous Variable | Shapiro-Wilk W Value | P Value | Distribution Status |
| --- | --- | --- | --- |
| Age (years) | 0.987 | 0.62 | Normal |
| Body Mass Index (kg/m²) | 0.982 | 0.45 | Normal |
| Preoperative Hemoglobin (g/L) | 0.979 | 0.38 | Normal |
| Preoperative Serum Albumin (g/L) | 0.985 | 0.57 | Normal |
| Surgery Duration (min) | 0.976 | 0.31 | Normal |
| Time to Detection of Congested Flaps (h) | 0.892 | 0.02 | Non-normal |
| Time to Re-exploration (h) | 0.875 | 0.01 | Non-normal |
