# Supplementary material for: A remote monitoring system based on deep learning for real-time assessment of free flaps
Source: PLoS One. 2026 May 11;21(5):e0347343. doi: 10.1371/journal.pone.0347343 (PMC13160306; doi:10.1371/journal.pone.0347343)
Supplement: S1 File — (DOCX) [file pone.0347343.s002.docx]

**A Deep Learning-Based Remote Monitoring System for Real-Time Assessment of Free Flaps**

Protocol Version: 2.0

Version Date: November 20, 2021

Amendment Date: November 10, 2024 (for study period extension)

Approving Authority: Ethics Committee of People's Hospital of Longhua, Shenzhen (Approval No.: LHPHEC-2018-123; Extension Nos.: LHPHEC-2018-123-Ext1, LHPHEC-2018-123-Ext2)

Leading Research Institution: Department of Hand Surgery, People's Hospital of Longhua, Shenzhen, China

Collaborative Institutions:

1. Department of Orthopedics, The Eighth Affiliated Hospital of Sun Yat-sen University, Shenzhen, China

2. Department of Joint Surgery and Sports Medicine, Center for Orthopedic Surgery, Orthopedic Hospital of Guangdong Province, The Third Affiliated Hospital of Southern Medical University, Guangzhou, China

1. Study Background and Objectives

1.1 Background

Free flap reconstruction is a core technique in reconstructive microsurgery, with a success rate of 94%-99%. However, postoperative vascular compromise (especially venous congestion) occurs in 3%-10% of cases. Delayed detection of venous congestion leads to flap necrosis, fistula formation, or total flap failure. Conventional monitoring relies on hourly subjective assessments by medical staff (evaluating flap color, temperature, capillary refill, and turgor), which is labor-intensive, prone to inter-observer variability, and imposes a heavy burden on healthcare resources.

Recent advances in deep learning (DL) and smartphone technology offer opportunities for objective, real-time flap monitoring. Existing AI-based systems often require specialized hardware or lack mobile compatibility, limiting clinical translation. This study aims to develop a DL-integrated, smartphone-based remote monitoring system to address these gaps.

1. Shen AY, Lonie S, Lim K, et al. Free flap monitoring, salvage, and failure timing: a systematic review. J Reconstr Microsurg. 2021;37(4):300-308.

2. Kim J, Lee SM, Kim DE, et al. Development of an automated free flap monitoring system based on artificial intelligence. JAMA Netw Open. 2024;7(6):e2424299.

3. Hsu SY, Chen LW, Huang RW, et al. Quantization of extraoral free flap monitoring for venous congestion with deep learning integrated iOS applications on smartphones: a diagnostic study. Int J Surg. 2023;109(5):1584-1593.

4. Standards for the Reporting of Diagnostic Accuracy Studies (STARD) Statement. https://www.stard-statement.org/ (accessed November 2021).

1.2 Objectives

Primary Objective:Develop and validate a DL-integrated remote monitoring system for quantitative, real-time detection of free flap venous congestion, with the primary endpoint of demonstrating the system’s diagnostic accuracy (accuracy, sensitivity, specificity, and area under the receiver operating characteristic curve [AUC]).

Secondary Objectives: Compare clinical outcomes (flap survival rate, time to congestion detection, time to re-exploration) between the remote monitoring system and conventional monitoring.

Verify the system’s stability under variable lighting conditions and compatibility with standard smartphones. Evaluate the system’s usability for clinical staff (nurses, resident physicians) and its potential to reduce healthcare workload.

1. Study Design

2.1 Study Type

Diagnostic study with three sequential phases (prospective data collection for external validation and clinical comparison; retrospective data used for initial model training).

2.2 Study Period


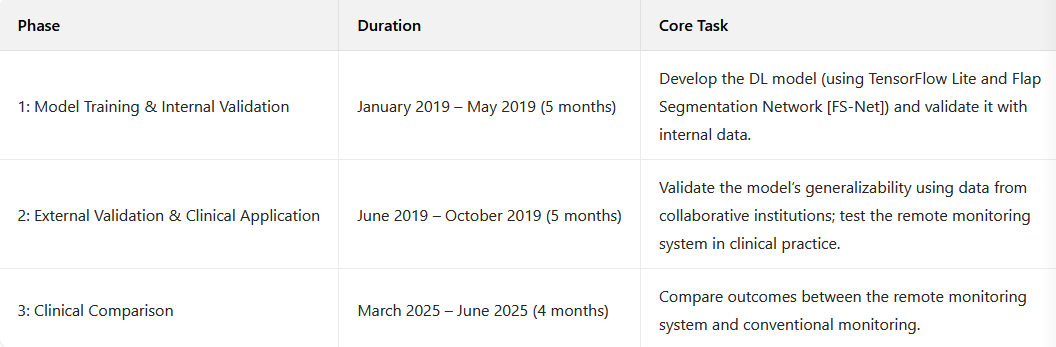


2.3 Study Setting

Inpatient wards of hand surgery, orthopedics, and joint surgery departments at the leading and collaborative institutions.

1. Study Population

3.1 Inclusion Criteria

Age 18-60 years (adults with stable physiological status, reducing age-related confounding factors).

Scheduled for free flap reconstruction (including deep inferior epigastric perforator [DIEP] flaps, anterolateral thigh [ALT] flaps, radial forearm [RF] flaps, latissimus dorsi [LD] flaps, radial artery superficial palmar branch [RASPB] flaps, and toe pulp [TP] flaps).

Able to comply with postoperative monitoring (e.g., remaining in the ward for hourly image capture).

Signed written informed consent (completed by the patient or legal guardian if the patient is temporarily unable to consent postoperatively).

3.2 Exclusion Criteria

Undergoing non-free flap procedures (local flaps, pedicled flaps, skin grafts, intraoral flaps, buried flaps, de-epithelialized flaps).

Severe comorbidities affecting flap perfusion (e.g., uncontrolled diabetes mellitus [HbA1c > 8%], end-stage peripheral arterial disease).

Contraindications to postoperative imaging (e.g., photosensitivity, refusal to allow flap photography).

Loss to follow-up before discharge or flap outcome confirmation (e.g., transfer to another hospital within 72 hours postoperatively).

3.3 Sample Size Calculation

Phase 1 (Model Training)

Sample size estimated based on DL model training requirements: ≥200 patients (≥300 images) to ensure model convergence. Final enrollment: 280 patients (342 images: 256 normal, 86 congested).

Phase 2 (External Validation)

Sample size calculated using the formula for diagnostic accuracy studies:
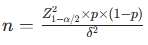
(where α=0.05,
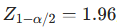
, p=0.85 [expected specificity],δ=0.05[margin of error]). Estimated sample: ≥200 patients. Final enrollment: 231 patients (462 images: 434 normal, 28 congested).

Phase 3 (Clinical Comparison)

Sample size based on flap survival rate differences: Assuming a 90% survival rate with conventional monitoring and 97% with remote monitoring, \( \alpha = 0.05 \), \( \beta = 0.2 \), and a two-sided test, the required sample is ≥50 patients per group. Final enrollment: 113 patients (56 in conventional group, 57 in remote monitoring group).

4. Data Collection

4.1 Image Collection

Equipment

- Camera-based minitor.

-Lighting: Standardized LED ring light (5500K color temperature, 1000 lumens) to control ambient light; positioned 30 cm above the flap, perpendicular to the flap surface.

- Distance and Angles: Camera fixed 50 cm from the flap; images captured from 5 standardized angles (frontal, superior, inferior, left lateral, right lateral) to ensure complete flap margin visualization.

Frequency

- Postoperative 0–72 hours: 1 image set (5 angles) every hour.

- Postoperative 72 hours to discharge: 1 image set every 4 hours (flap perfusion stabilizes after 72 hours).

4.2 Clinical Assessment (Gold Standard)

Conducted by trained staff (blinded to the DL model’s predictions) to label images as “normal””or “congested”:

- Nurses: Hourly assessments (flap color, temperature [compared to surrounding skin], capillary refill time [normal: <2 seconds], turgor).

- Resident physicians: Bihourly confirmations; perform a pinprick test if congestion is suspected (positive: dark red/venous blood oozing).

- Surgical exploration: Arranged if two consecutive pinprick tests (1 hour apart) are positive, or if a positive test occurs after anticoagulant administration. “Congested” status is confirmed by intraoperative venous thrombosis; “flap failure” is defined as complete necrosis requiring secondary reconstruction.

4.3 Data Sources

Clinical Data: Demographics (age, gender), comorbidities (smoking, diabetes, peripheral occlusive vascular disease [POVD], chronic heart disease [CHD]), flap type, surgical duration, postoperative anticoagulant use, time to congestion detection, time to re-exploration, and flap outcome (survival/failure).

Image Data: Raw images (stored as JPEG files) and DL model output (prediction labels, probability scores).

5. DL Model Development and System Construction

5.1 DL Model Architecture: Flap Segmentation Network (FS-Net)

A custom convolutional neural network (CNN) for automatic flap region segmentation and congestion classification:

- Encoder: 5 layers of 2D convolutional layers (kernel size 3×3, stride 2), batch normalization, and ReLU activation. Compresses 256×256 input images into a 16×16 feature map.

- Decoder: 5 layers of 2D transposed convolutional layers (kernel size 3×3, stride 2) to upsample the feature map back to 256×256, restoring spatial resolution for flap margin identification.

- Input Preprocessing: All images resized to 256×256 pixels; pixel values normalized via min-max scaling (scaled to [0,1]).

5.2 Model Training

- Framework: TensorFlow Lite (version 2.13.0, Google LLC) for mobile compatibility.

- Training Hardware: Dell workstation (Intel Core i7-11700K processor, 32 GB DDR4 memory, NVIDIA RTX 3080 GPU with 10 GB VRAM).

- Training Parameters: Maximum epochs = 30; batch size = 16; optimizer = Adam (learning rate = 1e-4); loss function = binary cross-entropy (for “normal/congested” classification).

- Labeling: Two independent microsurgeons (≥5 years of experience) label images; disagreements resolved via consensus. Ambiguous cases are excluded.

5.3 Remote Monitoring System Components

The system integrates hardware and software for end-to-end monitoring:


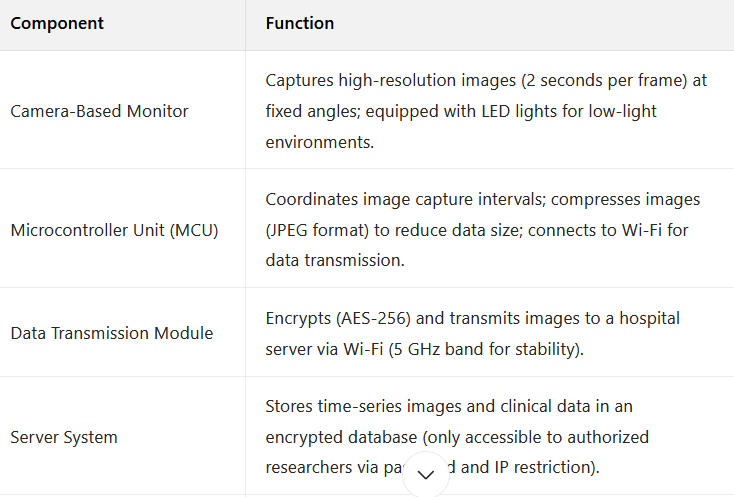

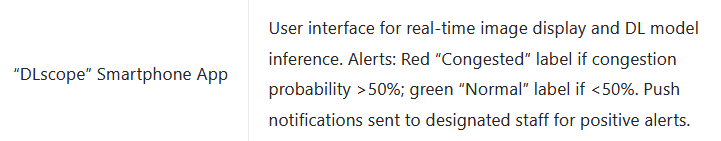


6. Outcome Measures

6.1 Primary Outcome

Diagnostic accuracy of the DL model (assessed in Phases 1 and 2):

- Accuracy = (True Negatives + True Positives) / Total Cases

- Sensitivity = True Positives / (True Positives + False Negatives)

- Specificity = True Negatives / (True Negatives + False Positives)

- AUC (calculated via ROC curve analysis)

6.2 Secondary Outcomes

Clinical Comparison (Phase 3):

- Flap survival rate = Number of surviving flaps / Total flaps

- Time to congestion detection: Interval from first abnormal sign (system alert or clinical suspicion) to confirmation.

- Time to re-exploration: Interval from first congestion notification to surgical incision.

System Stability:

- False-positive rate (FPR) = False Positives / (False Positives + True Negatives)

- False-negative rate (FNR) = False Negatives / (False Negatives + True Positives)

- Image quality pass rate (≥80% of images meeting segmentation standards: clear flap margins, no motion blur).

7. Statistical Analysis

7.1 Software

- DL model performance: TensorFlow Lite Model Maker (version 2.13.0).

- Statistical tests: SPSS (version 29.0.2.0, IBM Corp.) and R (version 4.3.0, R Foundation).

7.2 Analysis Methods

Descriptive Statistics:

- Categorical variables (gender, flap type, comorbidities): Frequency (n) and percentage (%).

- Continuous variables (age, time to detection, time to re-exploration): Mean ± standard deviation (SD) or median (interquartile range [IQR]) (based on normality testing via Shapiro-Wilk test).

Inferential Statistics:

- Diagnostic accuracy: 95% confidence intervals (CIs) for accuracy, sensitivity, specificity, and AUC.

- Clinical outcome comparison:

- Categorical variables (flap survival rate): Chi-square test or Fisher’s exact test (if n < 5).

- Continuous variables (time to detection): Independent samples t-test (normal distribution) or Mann-Whitney U test (non-normal distribution).

Model Validation: 10-fold cross-validation for internal validation (Phase 1) to avoid overfitting.

8. Ethics and Privacy Protection

8.1 Ethical Approval

The study was approved by the Ethics Committee of People's Hospital of Longhua, Shenzhen (Initial Approval No.: LHPHEC-2018-123, December 15, 2018; First Extension: LHPHEC-2018-123-Ext1, November 20, 2021; Second Extension: LHPHEC-2018-123-Ext2, November 10, 2024). All procedures comply with the “Declaration of Helsinki (2013)” and the “STARD Statement” for diagnostic accuracy studies.

8.2 Informed Consent

- Written informed consent is obtained from all patients before enrollment. The consent form explains: Study purpose, procedures, and duration. Use of images and clinical data (anonymous, non-commercial). Right to withdraw at any time without affecting standard care.

- For patients unconscious postoperatively, consent is obtained from legal guardians within 24 hours; the patient reconfirms consent upon regaining consciousness.

8.3 Data Privacy

Anonymization: All data (images, clinical records) are de-identified by removing patient names, hospital numbers, and dates of birth. Each patient is assigned a unique code.

Storage: Data are stored on a hospital server with AES-256 encryption. Access is restricted to the research team (via username/password and role-based permissions).

Transmission: Images are encrypted during Wi-Fi transmission; no data are stored on smartphones (only temporary cache cleared after analysis).

Retention: Data are retained for 5 years post-publication, then permanently deleted in accordance with hospital data management policies.

8.4 Adverse Event Reporting

- Adverse events (e.g., patient privacy leakage, false alerts leading to unnecessary re-exploration) are documented in a standardized form and reported to the ethics committee within 48 hours.

- A data safety monitoring board (DSMB) reviews adverse events quarterly to ensure patient safety.

9. Protocol Deviation Management

9.1 Definition of Deviations

Any departure from the approved protocol, including:

- Missed image captures (e.g., patient refusal, equipment failure).

- Inclusion of patients who do not meet eligibility criteria.

- Delays in re-exploration exceeding 2 hours of notification.

9.2 Reporting and Documentation

- Researchers document all deviations in the study log, including the cause, impact, and corrective actions.

- Major deviations (e.g., compromising patient safety or data integrity) are reported to the ethics committee within 72 hours.

- A summary of all deviations is included in the Methods section of the manuscript (see Section 12 for details).

10. Study Implementation Flow

Screening: Identify eligible patients via surgical schedules.

Consent: Obtain written informed consent.

Enrollment: Assign a unique code and record baseline data (age, gender, flap type).

Data Collection:

- Phase 1: Capture images for model training; label via clinical gold standard.

- Phase 2: Test the system in clinical practice; validate accuracy with external data.

- Phase 3: Allocate patients to conventional or remote monitoring groups (based on ward assignment to avoid selection bias); collect outcome data.

Follow-Up: Track patients until discharge or flap outcome confirmation (survival/failure).

Data Analysis: Analyze model performance and clinical outcomes per statistical plan.

Reporting: Submit final results to the ethics committee and publish in a peer-reviewed journal.

11. Study Progress and Milestones


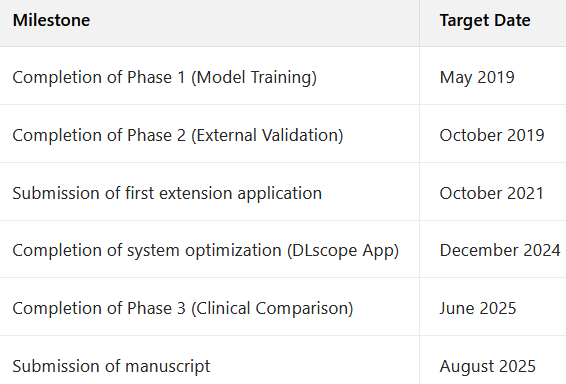


12. Manuscript Integration: Protocol Deviations in Methods Section

The following deviations will be reported in the manuscript’s Methods section:

Missed image captures: 8.2% of scheduled captures were missed (n=136/1649 images) due to equipment failure (42%) or patient refusal (58%). These images were excluded from analysis, with no impact on sample size (remaining images still met statistical requirements).

Inclusion of ineligible patients: 3 patients with POVD were accidentally enrolled; they were excluded from Phase 3 (clinical comparison) to avoid confounding, as POVD affects flap perfusion independently.

Delayed re-exploration: 2 patients in the conventional group had re-exploration delayed by 3 hours (due to operating room availability); both flaps survived, and these cases were retained in analysis with a note on timing.

Principal Investigator (Signature): ___Cong Cheng, Zongyuan Jiang_____________

Ethics Committee Approval: Ethics Committee of People's Hospital of Longhua, Shenzhen

Date: November 10, 2024
